# Comparison of visual scoring and planimetry methods for estimation of global infarct size on delayed contrast enhanced MRI and confrontation with biochemical markers of infarction

**DOI:** 10.1186/1532-429X-11-S1-P11

**Published:** 2009-01-28

**Authors:** Nathan Mewton, Pierre Croisille, Michel Ovize, Didier Revel

**Affiliations:** 1 Hopital Cardiovasculaire Louis Pradel, Lyon, France; 2 Hôpital Cardiovasculaire Louis Pradel, Lyon, France

**Keywords:** Acute Myocardial Infarction, Creatine Kinase, Infarct Size, Acute Myocardial Infarction, Reperfusion Therapy

## Introduction

Infarct size assessment by delayed enhanced CMR is of critical importance for the patient's prognosis after an acute myocardial infarction (AMI) and to assess the efficiency of new reperfusion therapies. However infarct size quantification by planimetry is time-consuming and difficult to do on a daily clinical practice.

## Purpose

By summing all the segmental scores using a 17-segment model, a global index of the size of the infarcted myocardium is easily obtained and we compared it to infarct size obtained by visual planimetry.

## Materials and methods

101 patients admitted with reperfused AMI to our intensive care unit were prospectively scanned using an ECG-gated gradient echo sequence after injection of gadolinium contrast agent. The global score was defined as the sum of the scores on each segment, and expressed as a percentage of the maximum possible score. This index was compared with a planimetric evaluation of hyperenhancement, expressed as a percentage of the left ventricle myocardial volume. The area under the curve (AUC) and peak values of serum troponin I (TnI) and creatine kinase (CK) release were measured in each patient.

## Results

There was an excellent correlation between visual planimetry and visual global scoring for the hyperenhancement extent's measurement (r = 0.91; y = 1.07x+2.3; SEE = 1.2; P < 0.001) The Bland-Altman plot shows a good concordance between the two approaches (mean of the differences = -3.9% with a standard deviation of 6.6). The mean percentage of hyperenhanced myocardium determined by the visual planimetry method was 21.2 ± 14.1% (median of 18.7%).

Mean post-processing time for visual planimetry was significantly longer than visual scoring post-processing time (23.7 ± 5.7 minutes VS 5.0 ± 1.1 minutes respectively, P < 0.001).

Correlation between CK AUC and visual planimetry was r = 0.73 (P < 0.001) and r = 0.77 (P < 0.001) with visual global scoring. Correlation between peak CK and visual planimetry was r = 0.72 (P < 0.001) and r = 0.77 (P < 0.001) with visual global scoring. Correlation between troponin I AUC and visual planimetry was r = 0.72 (P < 0.001) and between peak troponin I and visual planimetry was r = 0.56 (P < 0.001). Correlation between troponin I AUC and visual global scoring was r = 0.73 (P < 0.001) and between peak troponin I and visual global scoring was r = 0.59 (P < 0.001).

## Conclusion

A visual approach based on a 17-segment model can be used to evaluate the global myocardial extent of the hyperenhancement with similar results to planimetry and with shorter post-processing times.

